# Etiologies of Childhood Hearing Impairment in Schools for the Deaf in Mali

**DOI:** 10.3389/fped.2021.726776

**Published:** 2021-11-29

**Authors:** Abdoulaye Yalcouyé, Oumou Traoré, Abdoulaye Taméga, Alassane B. Maïga, Fousseyni Kané, Oluwafemi G. Oluwole, Cheick Oumar Guinto, Mohamed Kéita, Samba Karim Timbo, Carmen DeKock, Guida Landouré, Ambroise Wonkam

**Affiliations:** ^1^Faculté de Médecine et d'Odondostomatologie, Université des Sciences, Techniques et Technologies de Bamako (USTTB), Bamako, Mali; ^2^Division of Human Genetics, Department of Pathology, University of Cape Town, Cape Town, South Africa; ^3^Service de Neurologie, Centre Hospitalier Universitaire du Point “G”, Bamako, Mali; ^4^Service d'ORL, Centre Hospitalier Universitaire de Gabriel Touré, Bamako, Mali; ^5^Department of Medicine, University of Cape Town, Cape Town, South Africa

**Keywords:** hearing impairment, etiology, genetics, Mali, Africa

## Abstract

**Objectives:** To identify the etiologies of hearing impairment (HI) in schools for students who are deaf and to use a systematic review to summarize reports on the etiologies and clinical and genetic features of HI in Mali.

**Methods:** We included individuals with HI that started before the age of 15 years old. Patients were carefully evaluated under standard practices, and pure-tone audiometry was performed where possible. We then searched for articles published on HI in the Malian population from the databases' inception to March 30, 2020.

**Results:** A total of 117 individuals from two schools for the deaf were included, and a male predominance (sex ratio 1.3; 65/52) was noted. HI was pre-lingual in 82.2% (*n* = 117), and the median age at diagnosis was 12 years old. The etiologies were environmental in 59.4% (70/117), with meningitis being the leading cause (40%, 20/70), followed by cases with genetic suspicion (29.3%, 21/117). In 11.3% (8/117) of patients, no etiology was identified. Among cases with genetic suspicion, three were syndromic, including two cases of Waardenburg syndrome, while 15 individuals had non-syndromic HI. An autosomal recessive inheritance pattern was observed in 83.3% of families (15/18), and consanguinity was reported in 55.5% (10/18) of putative genetic cases.

**Conclusion:** This study concludes that environmental factors are the leading causes of HI in Mali. However, genetic causes should be investigated, particularly in the context of a population with a high consanguinity rate.

## Introduction

Hearing impairment (HI) is a partial or total inability to hear and is a leading cause of disability worldwide (1). Its global incidence is estimated to be 1/1,000 live newborns in developed countries, which is five to six times lower than that reported in developing countries (2, 3). This disparity is attributed to poor healthcare systems in developing countries, which are not always adequately equipped to prevent, screen, and manage the causes of HI (4). The prevalence varies widely from one region to another based on multiple factors, such as the prevalence of infectious conditions (meningitis, rubella, cytomegalovirus), and the quality of monitoring and assistance of pregnancy and birth (5). It affects both adults and children but has more serious implications among children due to its potential to interfere with language acquisition and cognitive development. At least 5% of the world's population, equivalent to 466 million people, experience a disabling HI and 34 million of these are children living in middle- and low-income countries (1). HI is a public health concern that negatively impacts people's well-being, with functional, economic, social, and emotional impacts.

HI can be classified based on several aspects, such as the age of onset, degree of hearing loss, location of the lesion, or etiology. The WHO reported that 60% of childhood HI is due to preventable causes (1). The etiology of HI can be environmental and/or genetic, and the prevalence of each group of etiologies varies across different regions. Environmental causes include infectious diseases (meningitis and measles), ototoxicity (medication), exposure to noise, trauma, or other factors such as low birth weight, prematurity, and neonatal jaundice. While infectious diseases are the leading causes of HI in low- and middle-income countries, their burden is lower in high-income countries (6–8). Some studies have reported that the etiology of almost 30% of HI remains unknown (9, 10). In developed countries, more than 50% of congenital HIs are of genetic origin (11–13).

Hereditary HI is divided into two main types: syndromic HI (SHI), where HI is associated with other organ abnormalities, and non-syndromic HI (NSHI), where HI is the only sign of disease. NSHI is the most common type of hereditary HI, accounting for ~70% of all hereditary HI types (11). HI is known to be associated with over 400 syndromes, including Waardenburg syndrome (WS), Branchio-Oto-Renal syndrome, Usher syndrome, Pendred syndrome, keratitis–ichthyosis–deafness syndrome, and Alport syndrome (4, 11, 14). The genetic cause of HI in numerous African countries, such as Mali, has not been properly investigated.

Only one study has assessed the etiologies of HI among 46 school-age children in Bamako, Mali. Meningitis was found as the leading cause (54.3%), while the etiology was not known in 19.6% of cases (10). Despite the high prevalence of HI in Mali (1), there are few data describing the etiology of this disease (10, 15, 16). Therefore, we performed the present study to identify the etiologies of HI among school-aged children in Mali, and to conduct a systematic review of previously reported data, emphasizing frequency, etiologies, and clinical and genetic features of HI in Mali.

## Materials and Methods

### Patients

The study was performed at the two schools for the deaf in Bamako, Mali and at the Department of Neurology of the Teaching Hospital of Point “G” (**Figure 2**), in collaboration with the Division of Human Genetics, Faculty of Health, University of Cape Town, Cape Town, South Africa. We included individuals with HI that started before the age of 15 years. Parents and patients were gathered at the schools for the deaf to explain the study objectives, emphasizing the interest in familial or non-environmental cases. The voluntary nature of participation and the possibility of withdrawing without consequences were highlighted. Families that were registered were called for enrollment in the following days. After obtaining informed consent, sociodemographic data and detailed medical information, including prenatal, neonatal, postnatal, and family history, were obtained from all participants.

### Clinical Assessment

Each patient completed a questionnaire and underwent a careful clinical examination. Pure tone audiometry was performed on all available patients who lacked audiological tests with a mobile audiometer (KUDUWAVE^TM^ N° 090103564). The audiological testing results obtained before school admission were reviewed for some patients. The hearing level was classified according to the International Bureau of Audiophonology number 02/1 (www.biap.org). For families with a suspected genetic origin of HI, a pedigree was drawn to elaborate on the patterns of inheritance. When SHI was suspected, additional tests, such as serum creatinine level, thyroid hormone levels, kidney and thyroid gland ultrasounds, temporal bone CT-scan, when possible, and ophthalmological assessment, were later performed to refine the diagnosis.

### Operational Definitions

In the context of the present study, HI was defined as: (1) acquired if there was a relationship between a putative environmental factor and the onset of the HI; (2) likely genetic when more than one case was reported in the family, in the case of consanguinity, and in clinically well-defined syndromic cases; and (3) of unknown etiology when the cause was not established as environmental or genetic, as previously reported by Wonkam et al. (9).

### Literature Review Process

#### Selection Criteria for the Literature Review

We included studies published from the databases' inception to March 30, 2020, that reported data on the prevalence, etiology, and clinical or genetic features of HI in the Malian population. In addition, specific Malian authors' names active in the field of HI were used to complement the literature searches. There were no restrictions on the reporting language of the article, and accessible full-length articles were selected.

#### Method of Search for Relevant Articles

We searched PubMed, Google Scholar, Microsoft Academy, Scopus, Science Direct, MEDLINE, African Journals Online, AFROLIB, and African Index Medicus, using the keywords “hearing impairment OR hearing loss OR deafness AND Mali.” As Mali is a major French-speaking country, the search was also conducted in French.

#### Ethical Considerations

This study was conducted in accordance with the guidelines of the Declaration of Helsinki and approved by the Institutional Ethics Committee of the Faculty of Medicine and Dentistry of the University of Sciences, Techniques and Technologies of Bamako, Mali (N°2020/129/CE/FMOS/FAPH), and the University of Cape Town (HREF REF: 691/2020). Informed consent was obtained from all the participants in this study.

## Results

### Clinical and Audiological Data

**Figure 1:**
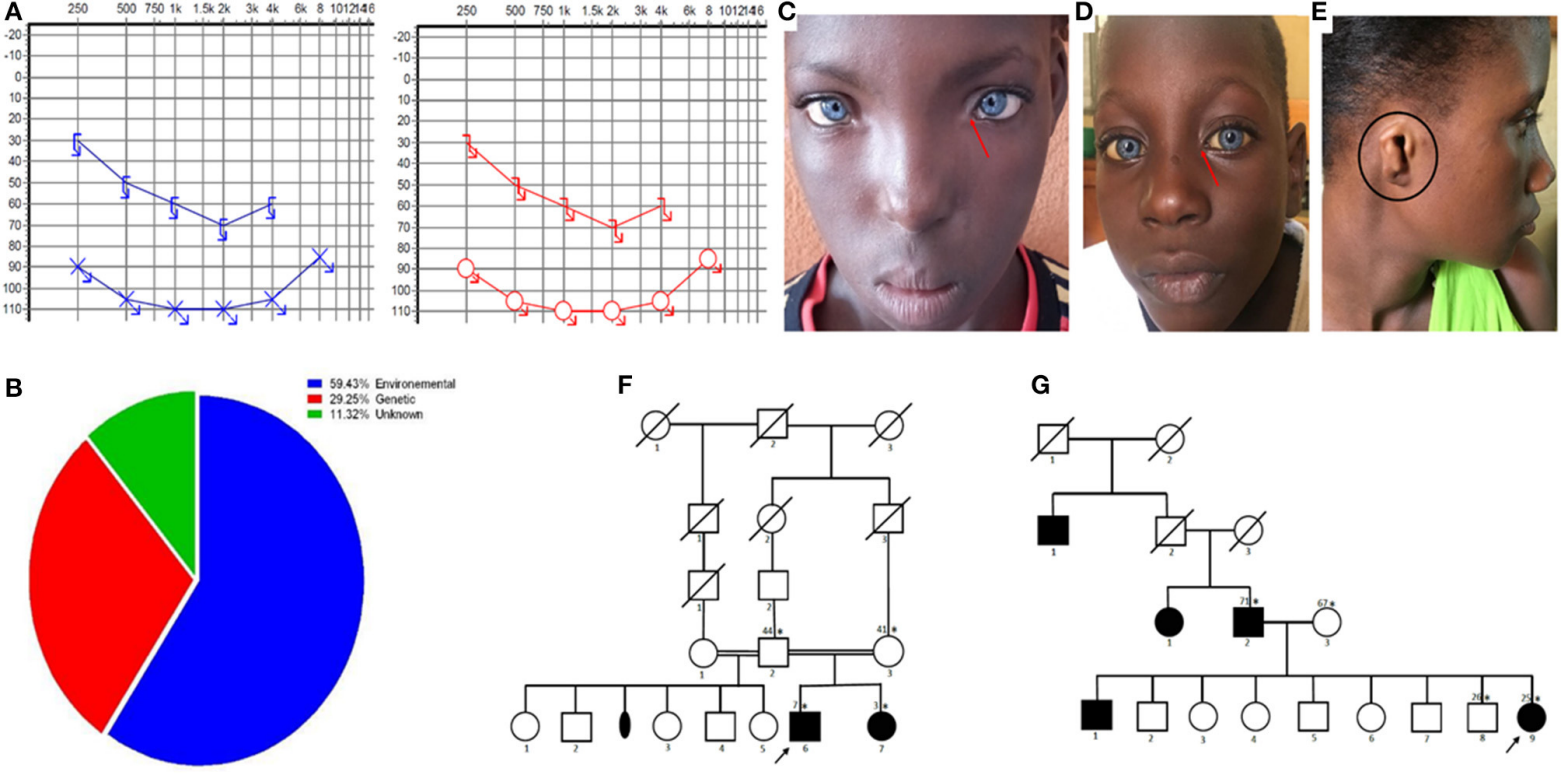
Clinical profiles. **(A)** Audiogram of a patient showing a bilateral and profound sensorineural hearing impairment (blue is the left side and red is the right side). **(B)** Diagram of the etiologies of hearing impairment in Mali. **(C,D)** Photos of patients with Waardenburg syndrome type 1 with dystopia canthorum (red arrow), and type 2 without dystopia canthorum (red arrow). **(E)** Photo of a patient with congenital microtia-deafness syndrome showing a right side microtia (black circle). **(F,G)** Pedigree of the families showing an autosomal recessive and autosomal dominant pattern of inheritance (asterisks represent individuals seen in clinic, numbers on the left are ages, and the arrow shows the proband).

We enrolled 100 families, totaling 117 individuals with HI, of whom 65 were male and 52 were female (sex ratio: 1.3; Table 1). Most participants belonged to the Bambara ethnic group from Kayes (the first region of Mali). Overall, 89.7% (105/117) of the participants were attending primary schools, and only 4.3% (5/117) were attending secondary schools. Seven patients were school attendant siblings who dropped out of school at the primary level. The median age at diagnosis was 12 years old, and HI was pre-lingual in 82.2% (96/117) of the individuals. Audiometry was performed in 61 participants and sensorineural HI was confirmed in 80.7% of the cases (49/61) (Table 1; Figure 1A). The etiology was environmental in most cases 59.4% (70/117) (Figure 1B), with meningitis being the major factor in 40% (28/70) of the cases, followed by chronic otitis in 18.6% (13/70), and low birth weight in 15.7 % (11/70*)*. Ototoxic medications, congenital infection, and neonatal asphyxia were implicated in 14.3% (10/70), 8.6% (6/70), and 2.8% (2/70), respectively. In four women, the ototoxic medication quinine hydrochloride was administered for up to 9 months during their pregnancy. Following pedigree analysis in our cohort, HI was observed in another sibling or a relative of the proband in 18 families, suggesting a genetic origin. Among them, three were SHI, including two cases of WS (Figures 1C,D) and one case of congenital microtia-deafness syndrome (Figure 1E); 15 had NSHI features. The Soninke ethnic group was the most represented in familial cases, comprising 33.3% (*n* = 18). The inheritance pattern was consistent with autosomal recessive inheritance in 83.3% (15/18) (Figure 1F), autosomal dominant in 5.6% (1/18) (Figure 1G), and likely sporadic in 11.1% of the families (2/18). Furthermore, parents reported consanguinity in 55.5% of the putative genetic cases (10/18). More pedigrees of familial cases are provided in the Supplementary Material 1. The sociodemographic and phenotypic characteristics of the patients are summarized in Table 1.

**Table 1:** Sociodemographic, phenotypic expression, and causes of HI.

**Characteristics**				***N* (%)**
Socio-demographics	Age (years)	Median (range): 12 (3–37)	
	Sex	Male		65 (55.5)
		Female		52 (45.5)
HI phenotypes	Onset	Pre-lingual		96 (82.2)
		Post-lingual		21 (17.8)
	Type	Sensorineural		49 (80.7)
		Conductive		3 (4.6)
		Mixed		9 (14.7)
HI causes	Environmental	Meningitis		28 (40)
		Chronic otitis		13 (18.6)
		Low birth weight		11 (15.7)
		Other^*^		18 (25.7)
	Likely genetics	Clinical expression	Syndromic	15 (83.3)
			Non-syndromic	3 (16.7)
		Likely inheritance pattern	Autosomal recessive	15 (83.3)
			Autosomal dominant	1 (5.6)
			Sporadic	2 (11.1)
		Consanguinity rate		10 (55.5)
	Unknown			13 (11.3)

*HI, hearing impairment*.

* *Other = Ototoxic medications, congenital infection, and neonatal asphyxia*.

### Literature Review Data

Following a review of the literature, we identified eight articles that met our selection criteria (any article reporting HI cases in the Malian population). All studies were performed in Bamako, the capital city of Mali. Only one study was conducted at a school for the deaf, while four others were performed in hospital settings. We report three case reports and five cross-sectional case series. None of them reported the prevalence or incidence of HI in Mali or the genetic contribution to HI in Mali. However, they reported environmental factors in 80% (37/46) of the participants, with meningitis contributing up to 54.3% (25/46) of HI in a study conducted in one of the schools for the deaf of Bamako (10). The main diagnostic tool used was pure tone audiometry in almost all studies. In a study of 200 individuals conducted at a teaching hospital, the audiometric profile varied from a mixed type in 43.4% (87/200) to sensorineural in 32.2% (64/200) of HI cases (16). Two clinical cases of WS, including one associated with cleft lip and another associated with dystopia canthorum, have been reported (15, 17). One case of HI due to suspected ototoxic medication has been reported (18). Descriptions of these studies are summarized in Table 2.

**Table 2:** Characteristics of studies included in the literature review.

**References**	**Area**	**Region**	**Study setting**	**Study design**	**Males** *N* **(%)**	**Mean age (years)**	**Age range** **(years)**	**Sample size**	**Prevalence**	**Main etiologies**	**Diagnosis tool**
Mohamed et al. (10)	Urban	Bamako	School for the deaf	Cross-sectional cases series	30 (65.2)	11	5–19	46	NR	Meningitis	PTA
Traoré et al. (15)	Urban	Bamako	Hospital	Case report	0	1.6	NA	1	NR	NI	PTA
Imperato and Imperato (17)	Rural	Bougouni	Community	Case report	1 (100)	13	NA	1	NR	NI	NA
Traoré et al. (18)	Urban	Bamako	Hospital	Case report	1 (100)	33	NA	1	NR	Toxic	PTA
Sako (19)	Urban	Bamako	Community	Cross-sectional cases series	NR	NR	10–60	147	NR	Meningitis	PTA
Baba (20)	Urban	Bamako	Hospital	Cross-sectional cases series	533 (64)	6	0–15	833	NR	Meningitis	PTA
Diarra et al. (16)	Urban	Bamako	Hospital	Cross-sectional cases series	120 (60)	37.2	15–83	200	NR	NR	PTA
Sacko et al. (21)	Urban	Bamako	Community	Cross-sectional cases series	68 (100)	32	30–55	68	NR	Noise	PTA
This study	Urban	Bamako	School for the deaf	Cross sectional case series	65 (55.6)	7	4–21	117	NR	Meningitis	PTA

*NR, not reported; NI, not identified; NA, not applicable; PTA, pure tone audiometry*.

## Discussion

### Context of the Research

Mali is a landlocked country located in the center of West Africa and is surrounded by seven other countries. With a territory of 1,220,190 km^2^ and a population of 15,302,000, the country has a young population; 47% are under 15 years of age (22). Mali is an ethnically and culturally diverse country, and its subpopulations have a long tradition of intra-ethnic and consanguineous marriages (23) that have particularly favored gene identification for numerous recessive conditions (24, 25). In a study of about 600 Malian students from different ethnic backgrounds, 27% reported consanguinity, including 17% with parental first cousin marriages (26). This results in homogeneous cluster populations with typical phenotypic characteristics and an increased prevalence of recessive disorders in some parts of the country. Moreover, while family-based genetic studies are often limited in high-income countries due to small sibships, the average fertility rate in Mali, which is over six births per woman, offers a unique opportunity to find new disease genes or variants that can then be studied in other populations (23, 25).

There are at least 14 different major ethnic groups in Mali, each speaking a different language. However, about 80% of Malians speak the national language of Bambara, which is also spoken in five neighboring countries (23). Genetic studies were introduced to Mali ~15 years ago, leading to the identification of numerous genetic variants for rare neurogenetic diseases, some of which present with characteristic features; these outcomes are notably favored by the structure of the Malian population (26, 27). Despite this, genetic and genomic studies in Mali have been relatively limited to select families in a specific specialty; hence, there is an underestimation and neglect of genetic diseases and the genetic contribution to common diseases (25). This may be due to social factors surrounding these diseases, limited resources that prevent patients from seeking care, and a lack of infrastructure and medical geneticists. The low literacy rate in Mali, as in many developing countries, may foster a low level of understanding of basic genetic concepts in the general population. For instance, Malians often consider genetic diseases to be a result of a bad fate, which leads to stigmatization (27). The knowledge, attitudes, and beliefs of Malian families with hereditary neurological disorders regarding genetic testing were assessed in a previous study. The results showed that, in general, the majority favored genetic testing and some gained knowledge from genetic counseling (27).

### Sociodemographic, Clinical, and Genetic Profiles

This study reports the main etiologies of HI among school-aged children based on an observational study in two schools for the deaf and describes a review of the literature, thereby providing an improved comprehensive understanding of the etiologies of HI in Mali to date. Similar to reports from other sub-Saharan Africa (SSA) countries, our study revealed a high proportion of environmental causes (9, 28), while emphasizing the likely contribution of genetic etiology of HI in Mali. This study also revealed a serious epidemiological gap, as no reports on the incidence and prevalence of HI were found, urging the need for larger and more in-depth studies to evaluate its burden in SSA countries (1). Indeed, as reported in the present study and in the literature, pre-lingual HI is the most common type of HI among children, with a relatively late diagnosis. This interferes strongly with language acquisition, as this period is crucial for harmonized psychosocial development (29). It is not surprising that the Bambara ethnolinguistic group was the most represented in the cohorts, as it is the main ethnic group in the Malian population, representing 34.1% (10). Similar to other reports from SSA countries (9, 10, 16, 28), males were predominant in this study. As previously reported elsewhere in Africa, compared to female children, males are often preferentially sent to schools for the deaf (28). This is still a detrimental gender bias that needs to be addressed in society. Environmental factors were the main etiologies of HI in Malian children, as reported in many studies conducted across developing countries, including across Africa (4, 8, 9, 28, 30). This can be associated with limited access to adequately equipped healthcare centers to assist and monitor pregnancy and birth, and to provide relevant information on the environmental risk factors for HI in the community (5). In addition, along with Cameroon (9), Mali is located in the African meningitis belt characterized by seasonal epidemics (8, 16, 19), resulting in a higher burden of meningitis-associated HI in Mali (Table 1) and in other African countries (9, 31). Meningitis has also been identified as a major cause of HI in other developing countries outside of Africa (4, 9, 10, 20, 30). Additional environmental factors, such as mumps, measles, prematurity, or neonatal nuclear jaundice, were also identified as etiologies in this study but were not reported to play an important role in HI in some SSA countries (9). However, this could be due to a lack of health records or limited access to diagnostic tools.

The genetic contribution to HI varies widely among countries. In this study, we suspected its contribution in one-third of the cases, similar to studies conducted in Cameroon (8). This is lower than that reported in high-income countries, where nearly 50% of congenital HIs have a genetic origin. This is likely due to better prevention and interventions that have contributed to reducing environmental hazards, including newborn screening for HI, the availability of comprehensive care, and easy access to molecular diagnosis facilities to confirm suspected genetic causes (32). Moreover, the lack of proper genetic investigation facilities in Mali and other SSA countries may lead to an underestimation of HI of genetic origin. To date, over 120 genes have been associated with HI (33), and it is estimated that 1% of the human genome is involved in the hearing process (34). From the first HI-associated gene identified in 1995 (35) to date, genes are continuously being discovered, and some cases with unknown etiologies could probably have a genetic component, as suggested by Fraser (36). The higher level of consanguinity in Mali (26) should be associated with a much higher rate of congenital HI of genetic origin. This was confirmed by the high autosomal recessive inheritance patterns observed in our cohort. In fact, most of the familial cases reported in this study were seen in the Soninke ethnic group, which has a high tendency for consanguineous marriage. In Mali, as in numerous understudied African populations, it is probable that numerous variants of known and potentially novel genes remain to be discovered (37–39). Among cases with a suspected genetic origin, we identified two families with WS, the most common SHI reported in some countries (8). WS is associated with a myriad of symptoms, including dystopia canthorum (lateral displacement of the inner canthus of each eye); pigmentary abnormalities of the hair, iris, and skin (often a white forelock and heterochromia iridis); and sensorineural deafness (33). One case presented with a type 1 WS with dystopia canthorum and the other presented with type 2 WS. Two other cases of WS were previously reported in the Malian population: type 1 associated with a labial cleft and a classical type 1 case with no other malformations (15, 17). Similar to other reports in SSA (9), we also found one case of congenital microtia associated with profound HI, confirming its rarity in the literature. Unsurprisingly, NSHI was the most common subtype among putative genetic cases in our study, confirming what is already known worldwide (11).

### Strengths and Limitations

**Figure 2:**
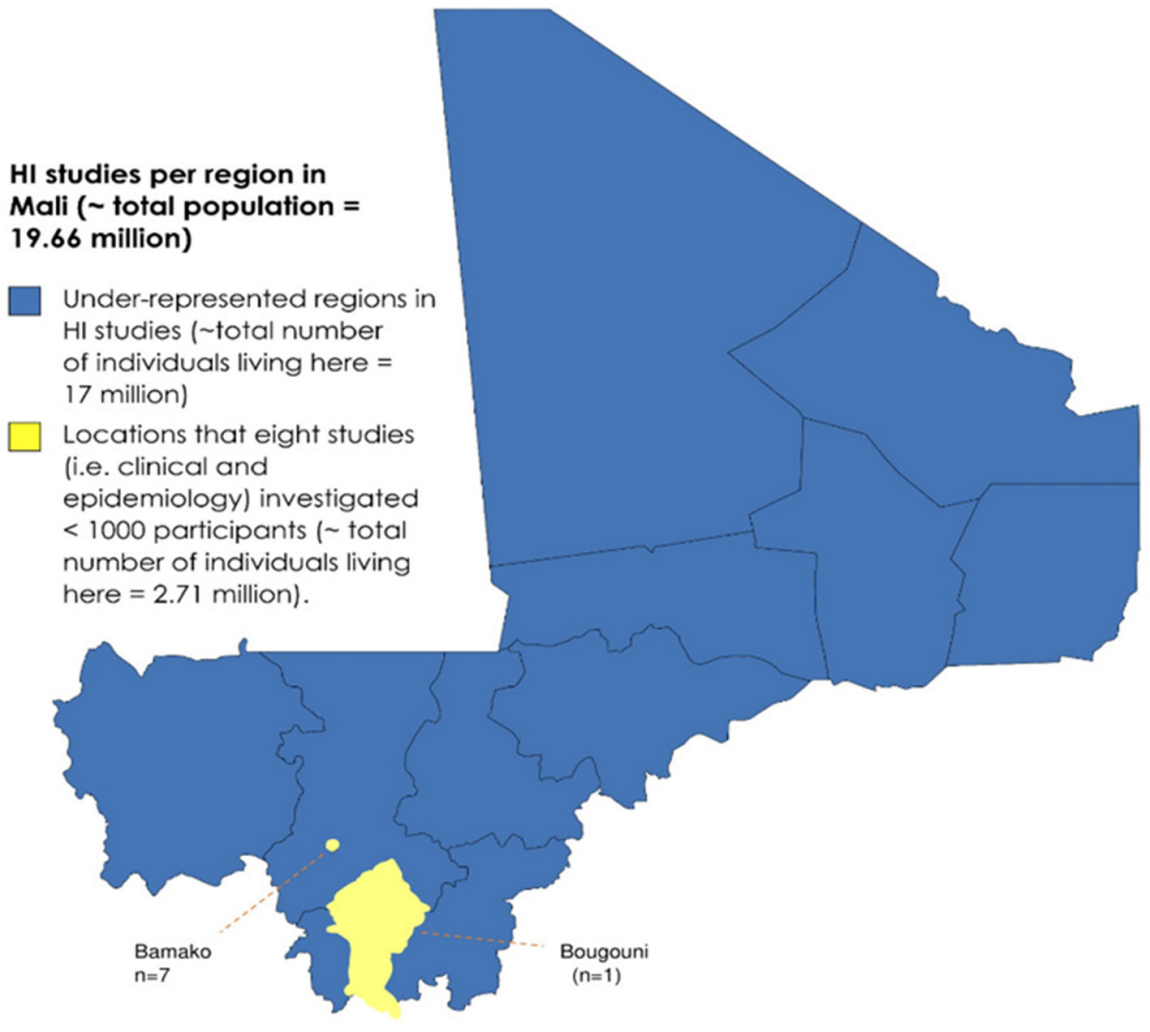
Map of Mali showing the regions where the studies in this review were performed. The geographical representation of HI studies in Mali to date.

The data presented here had some limitations. First, the recruitment from schools for the deaf cannot be representative of the entire country, as not all communities will have access to these schools (Figure 2). In addition, there is a lack of systematic screening for HI during the admission of children to normal schools in Mali due to the limited availability of audiologists and trained nurses in school clinics, and the limited knowledge of teachers and parents about the early detection of hearing-impaired children. These aspects can limit the clinical characterization of hearing-impaired individuals, even those attending schools for the deaf, with a possible underestimation of syndromic cases. Despite these limitations, this cohort is the largest reported in schools for the deaf in Mali to date and represents an initial step toward a proper epidemiological description and genetic characterization of HI in the general population in Mali.

## Conclusion

This study confirms that environmental factors are the leading etiologies of childhood HI in Mali and emphasizes the high contribution of consanguinity to genetic causes of childhood HI in Mali. Policy actions must be taken in terms of raising awareness for early treatment of otitis and reinforcing the existing immunization programs to vigorously fight these preventable factors and decrease the occurrence of HI in developing countries. Therefore, there is an urgent need to undertake genetic investigations. The increasing access to whole exome sequencing may identify variants in HI genes that favor early treatment of HI to allow a normal life for deaf children and improve counseling of people with disease traits in recessive settings. This could also provide an opportunity for novel HI gene discovery that will further our understanding and trigger future drug development for these diseases.

## Data Availability Statement

The raw data supporting the conclusions of this article will be made available by the authors, without undue reservation.

## Ethics Statement

This research was conducted according to the guidelines of the Declaration of Helsinki and approved by the Institutional Ethics Committee of the Faculty of Medicine and Dentistry of the University of Sciences, Techniques and Technologies of Bamako, Mali (N°2020/129/CE/FMOS/FAPH) and the University of Cape Town (HREF REF: 691/2020). Informed consent was obtained from all participants of this study. Written informed consent was obtained from the individual(s) AND/OR minor(s)' parents or legal guardian/next of kin] for the publication of any potentially identifiable images or data included in this article.

## Author Contributions

GL and AW: conceptualization, supervision, and funding acquisition. AY, OT, AT, AM, and GL: methodology. AY and FK: collected the data. AY: analyzed the data and writing the first draft. OO, CD, CG, MK, ST, GL, and AW: editing and reviewing the manuscript. All authors have read and agreed to the published version of the manuscript.

## Funding

This study was supported by the NIH (USA), grant number U01-HG-009716 to AW, the African Academy of Science/Wellcome Trust, grant number H3A/18/001 to AW, and grant number U01HG007044 to GL.

## Conflict of Interest

The authors declare that the research was conducted in the absence of any commercial or financial relationships that could be construed as a potential conflict of interest.

## Publisher's Note

All claims expressed in this article are solely those of the authors and do not necessarily represent those of their affiliated organizations, or those of the publisher, the editors and the reviewers. Any product that may be evaluated in this article, or claim that may be made by its manufacturer, is not guaranteed or endorsed by the publisher.
