# Supplementary material for: Etiologies of Childhood Hearing Impairment in Schools for the Deaf in Mali
Source: Front Pediatr. 2021 Nov 29;9:726776. doi: 10.3389/fped.2021.726776 (PMC8667071; doi:10.3389/fped.2021.726776)
Supplement: Supplementary file 1 [file Data_Sheet_1.PDF]

Supplementary material: Selected families segregating hearing impairment in Mali

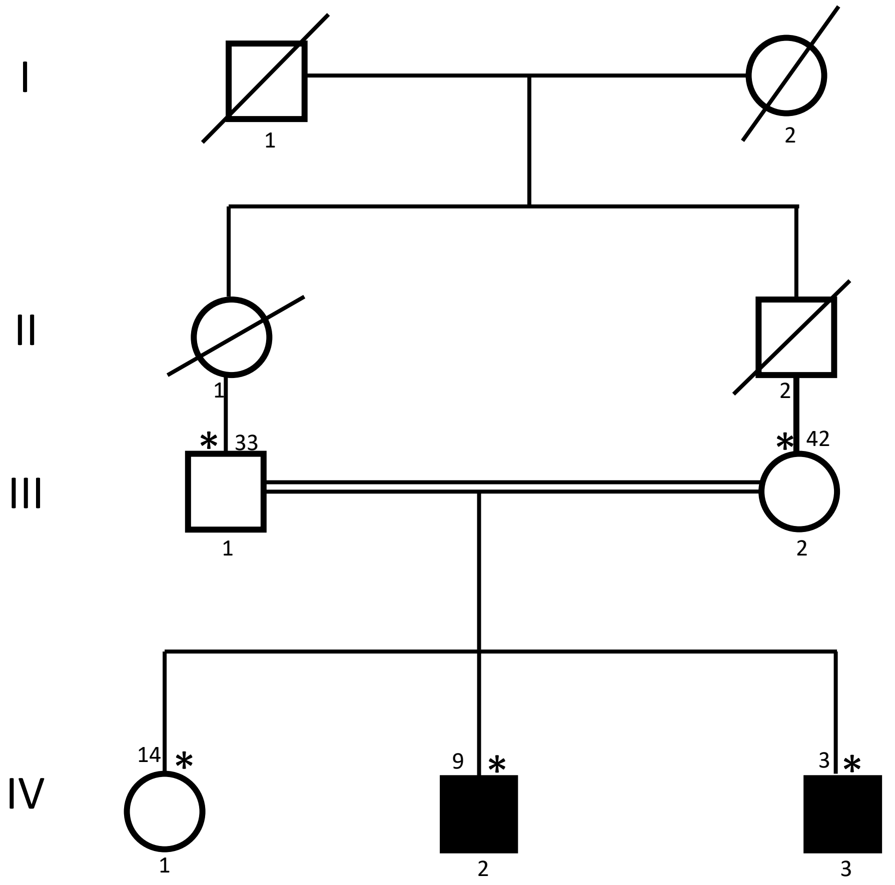

Pedigree of the family 3: Showing two affected individuals from consanguineous relationship

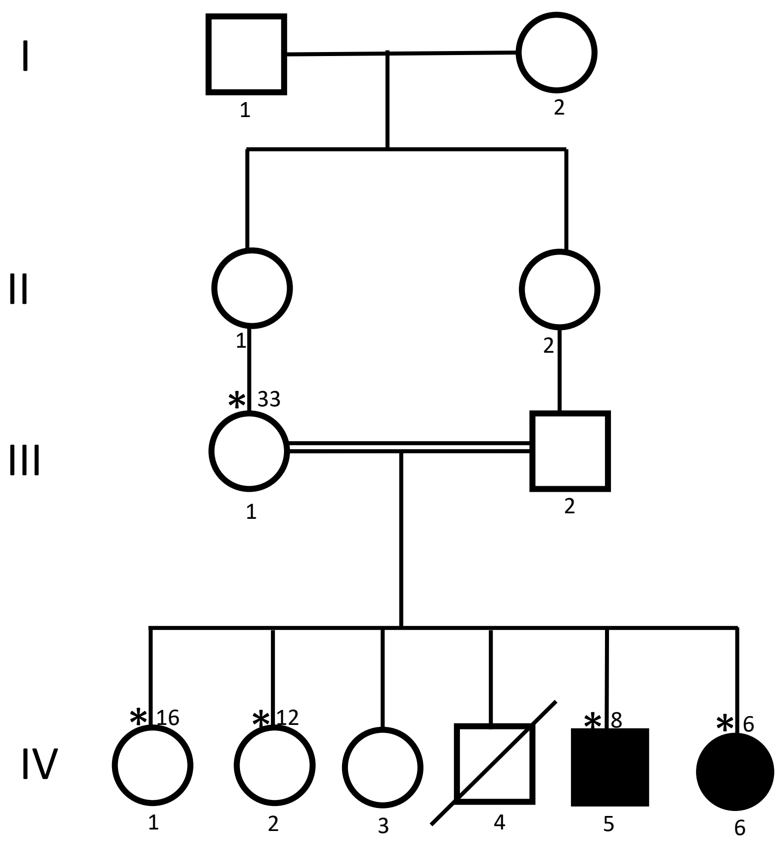

Pedigree of the family 4: Showing two affected individuals from consanguineous relationship

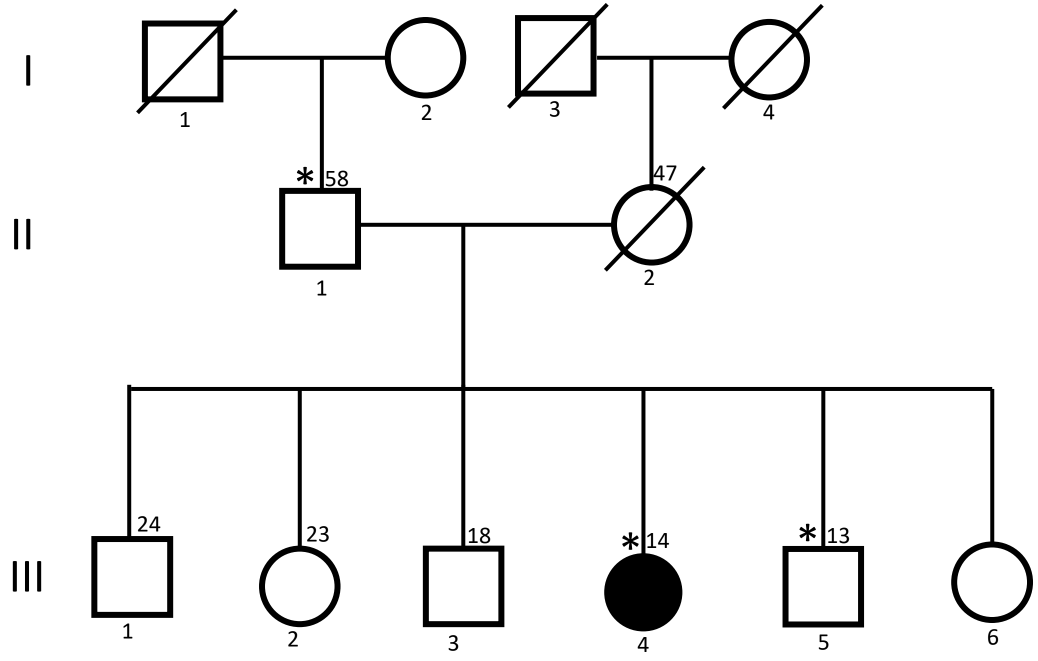

Pedigree of the family 5 with congenital microtia and deafness syndrome

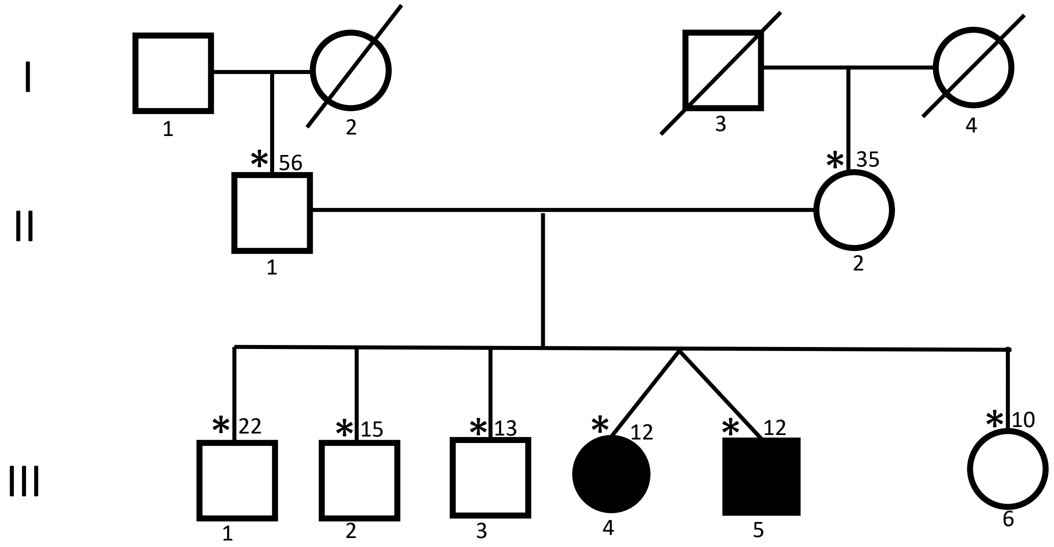

Pedigree of the family 6: Showing two affected individuals from non-consanguineous relationship

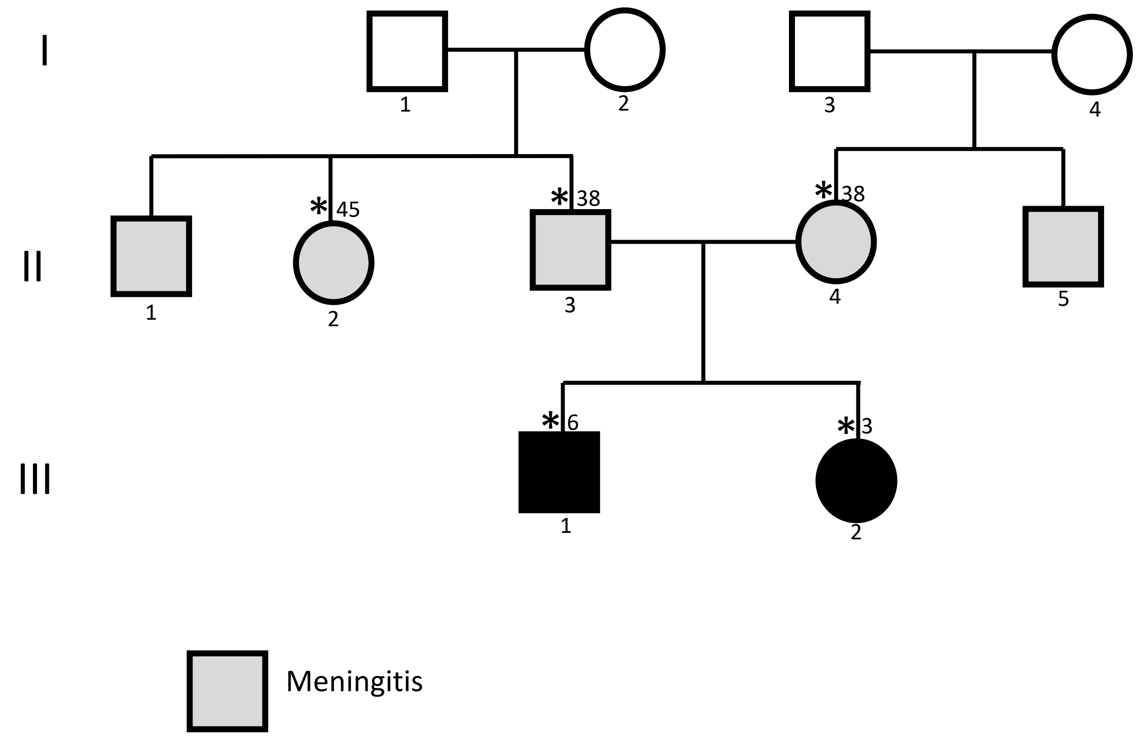

Pedigree of the family 7: Showing two affected individuals from non-consanguineous relationship

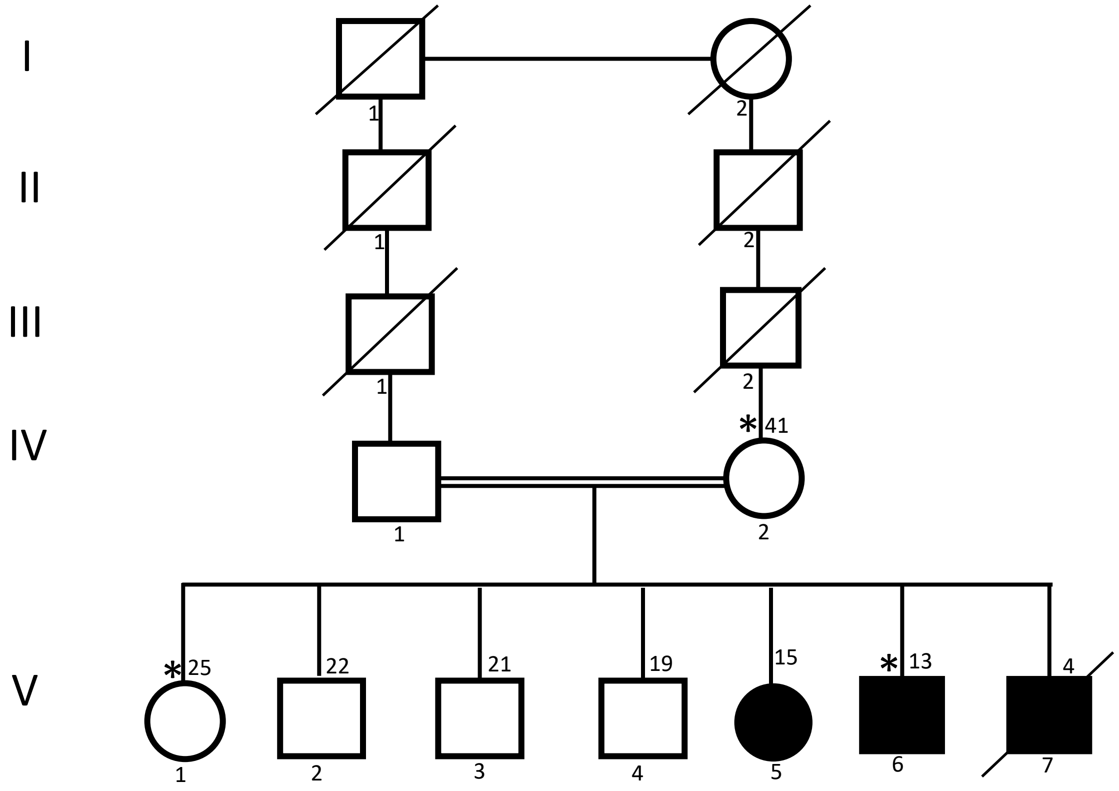

Pedigree of the family 8: Showing three affected individuals from consanguineous relationship

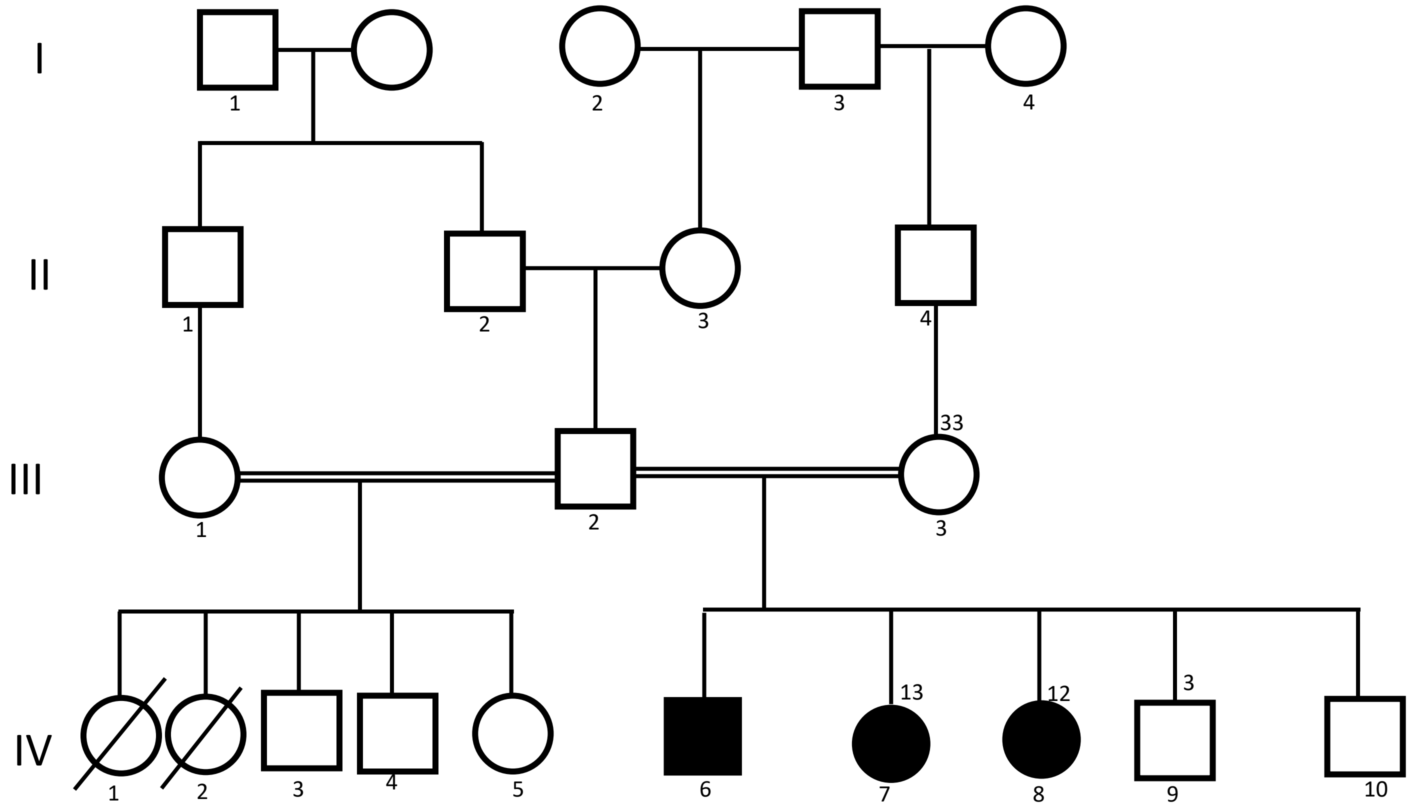

Pedigree of the family 9: Showing three affected individuals from consanguineous relationship

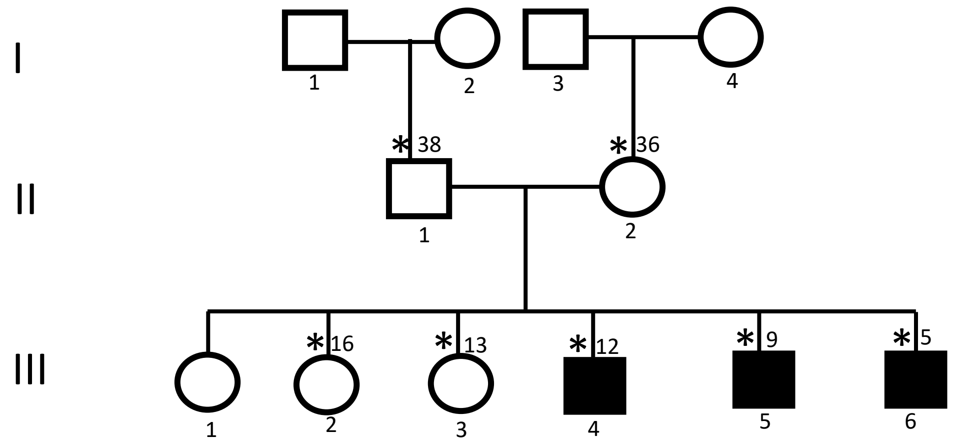

Pedigree of the family 10: Showing three affected individuals from non-consanguineous relationship

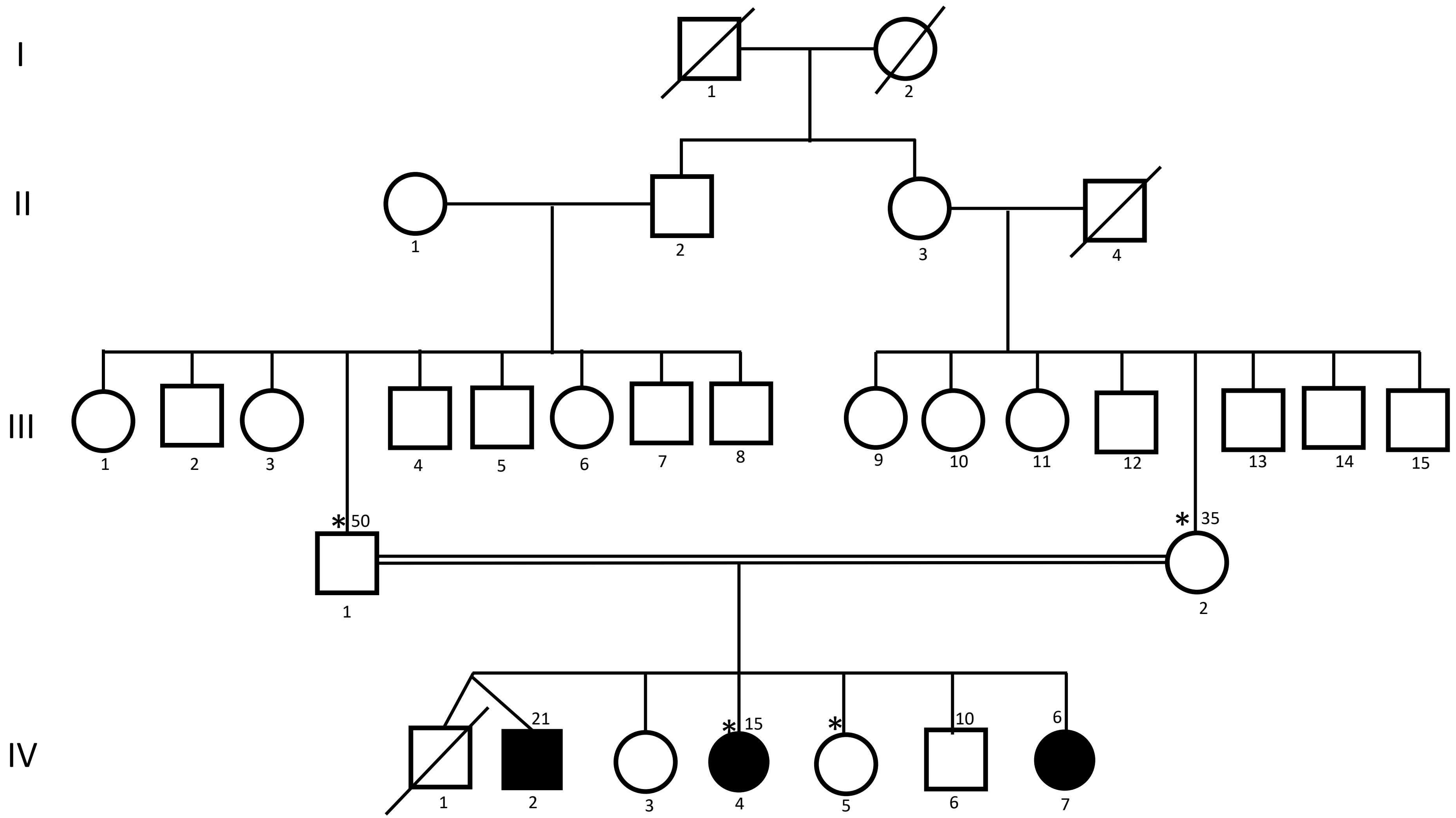

Pedigree of the family 11: Showing three affected individuals from consanguineous relationship

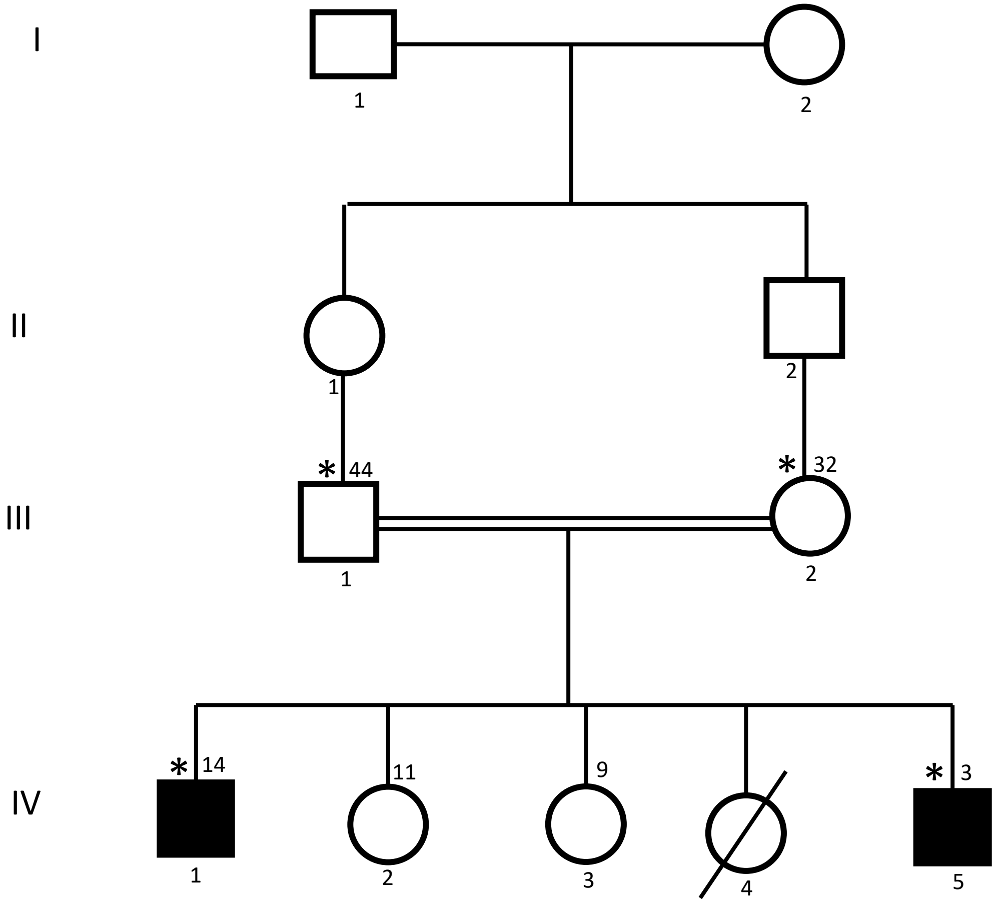

Pedigree of the family 12: Showing two affected individuals from consanguineous relationship

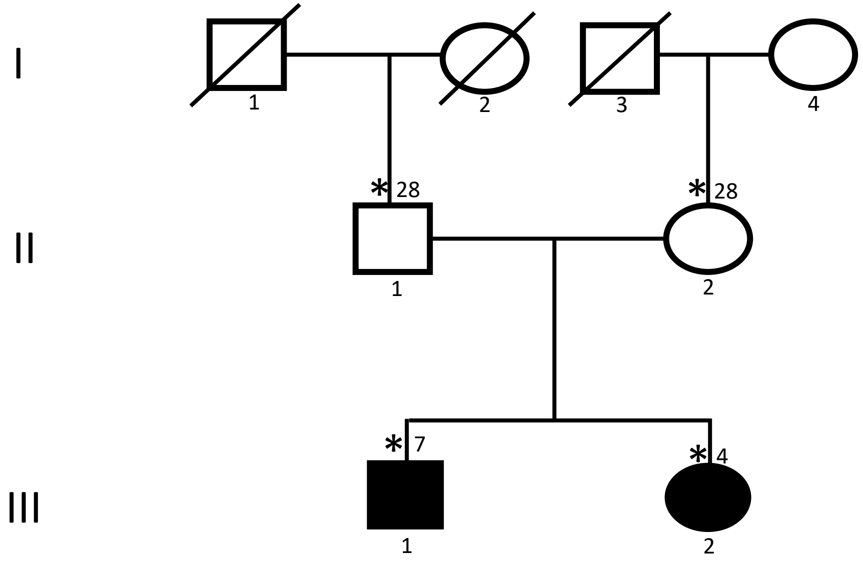

Pedigree of the family 13: Showing two affected individuals from non-consanguineous relationship

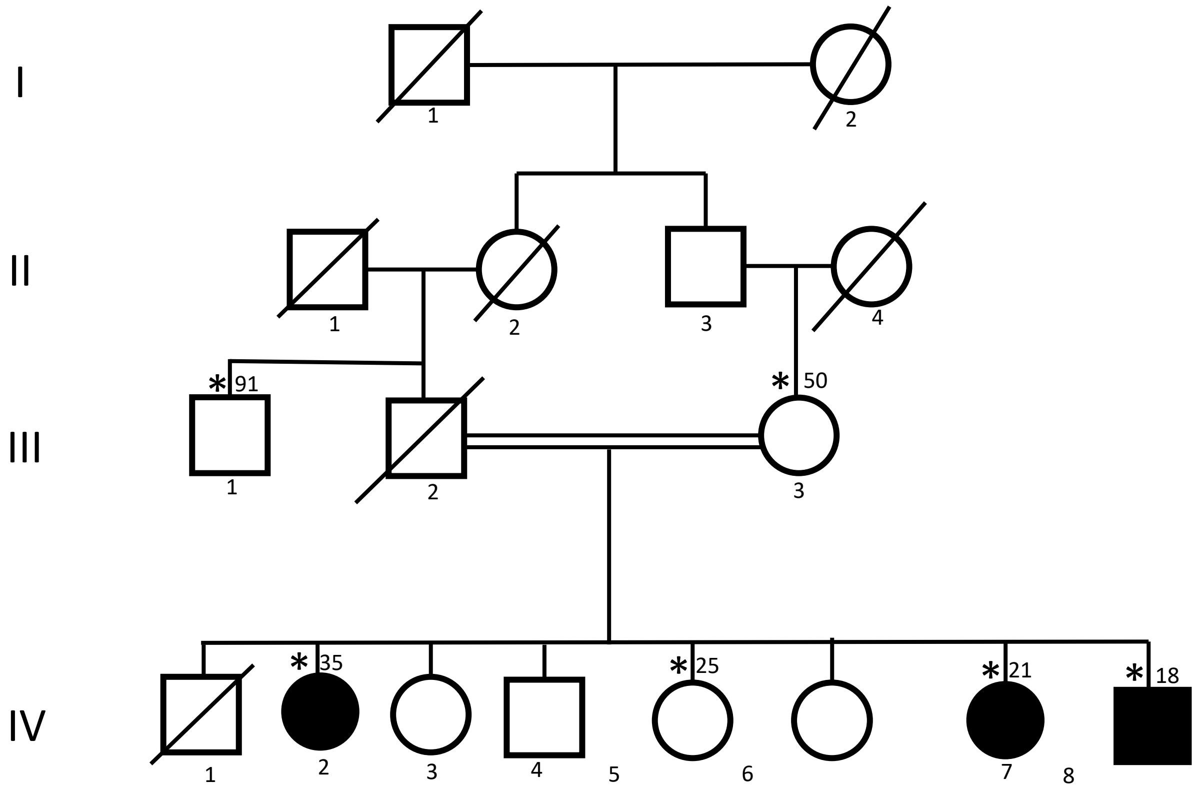

Pedigree of the family 14: Showing three affected individuals from consanguineous relationship

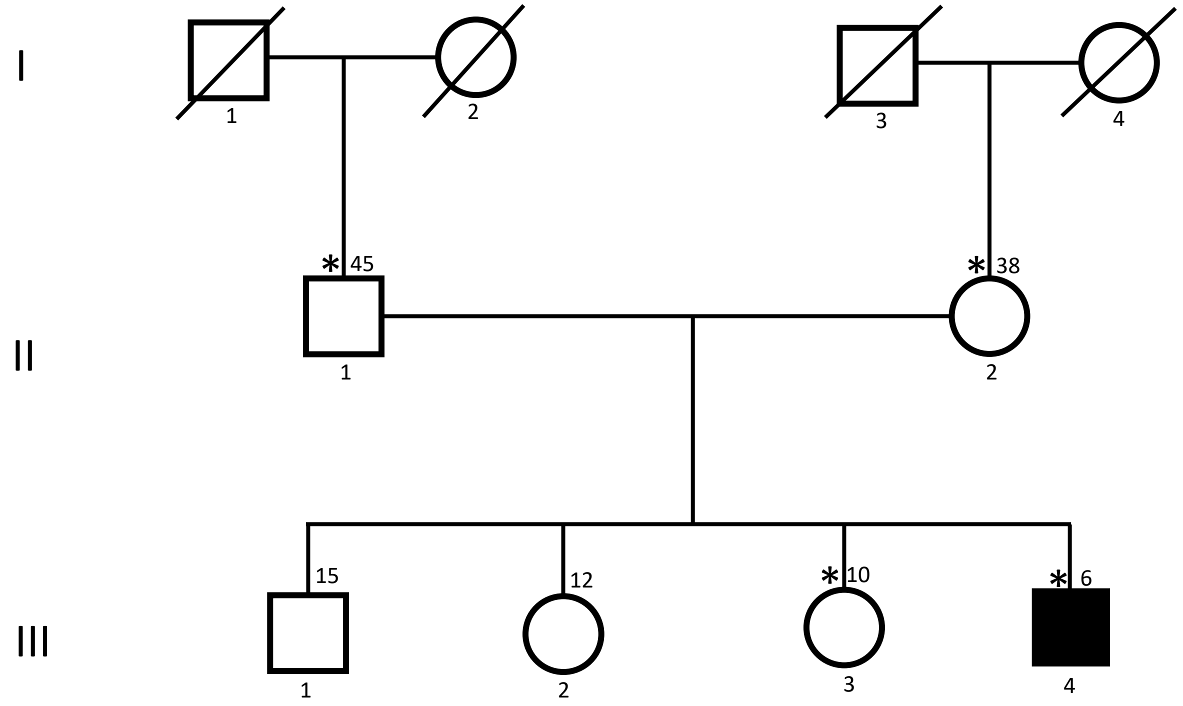

Pedigree of the family 15 with Waardenburg syndrome type 2

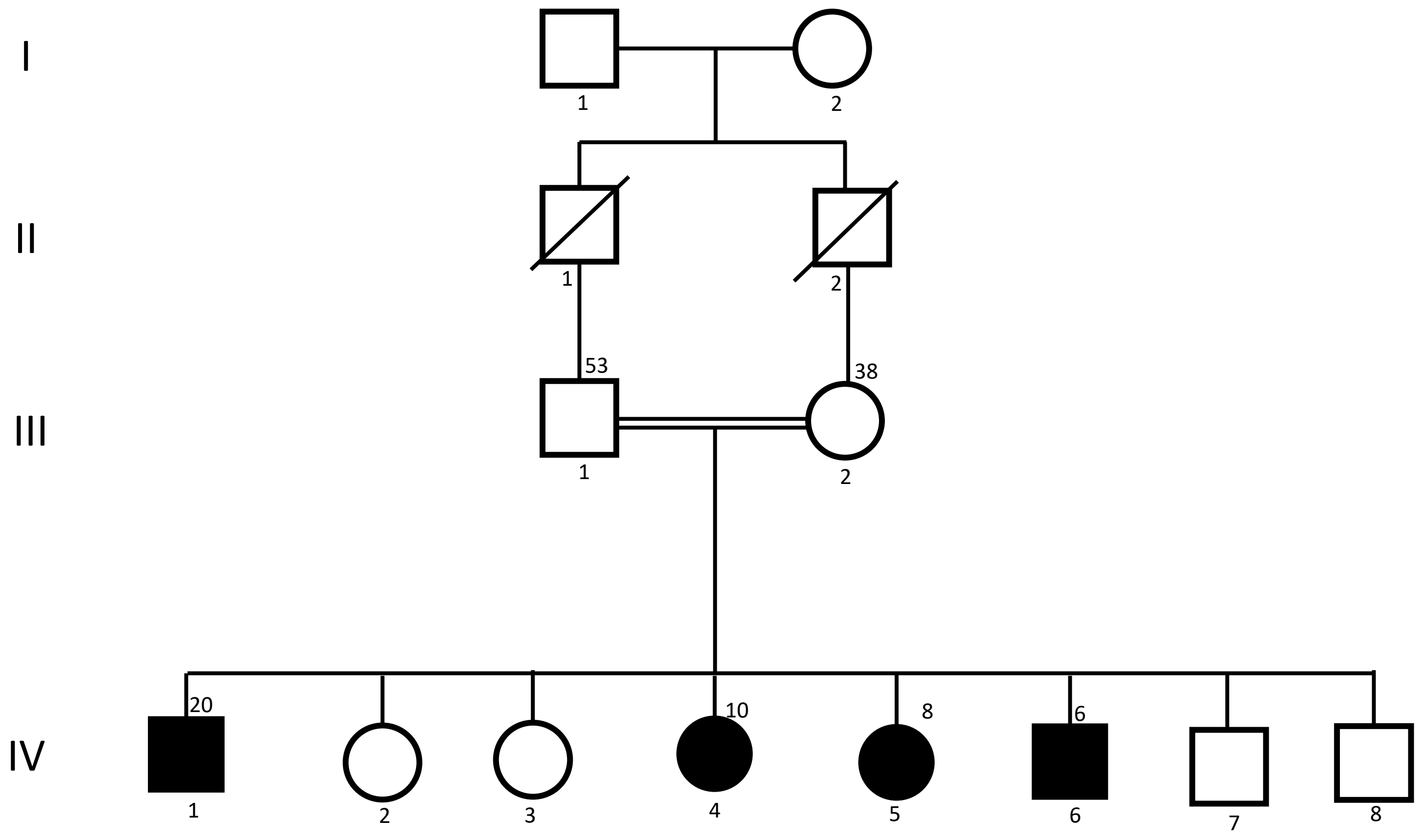

Pedigree of the family 16: Showing four affected individuals from consanguineous relationship

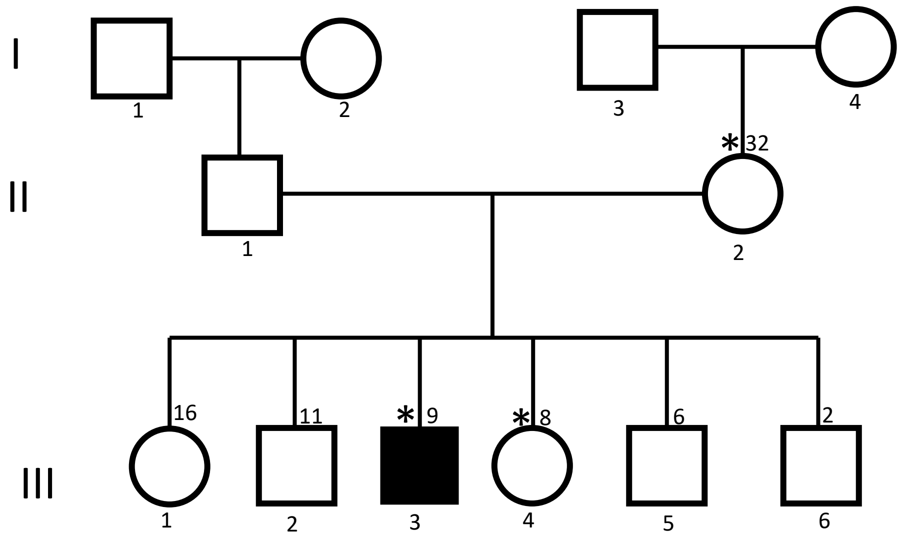

Pedigree of the family 17 with Waardenburg syndrome type 1

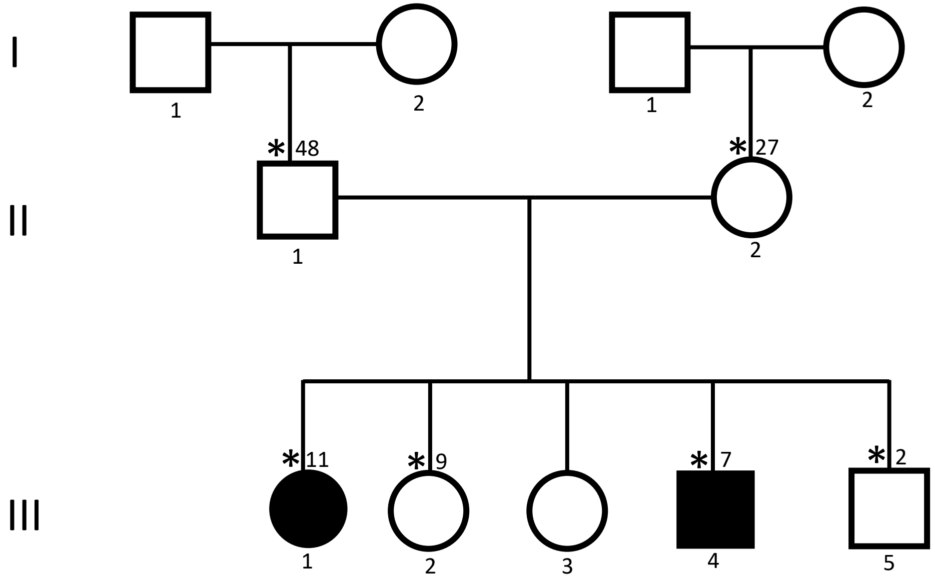

Pedigree of the family 18: Showing two affected individuals from non-consanguineous relationship
